# 2-(2-Naphth­yloxy)pyrimidine

**DOI:** 10.1107/S1600536809026592

**Published:** 2009-07-18

**Authors:** Nasir Shah Bakhtiar, Maizathul Akmam A. Bakar, Zanariah Abdullah, Seik Weng Ng

**Affiliations:** a Department of Chemistry, University of Malaya, 50603 Kuala Lumpur, Malaysia

## Abstract

In the title compound, C_14_H_10_N_2_O, the organic rings are inclined at an angle of 86.1 (1)°. The angle at the ether O atom is widened to 117.18 (14)°.

## Related literature

For 2-phenoxy­pyrimidine, see: Shah Bakhtiar *et al.* (2009 ▶).
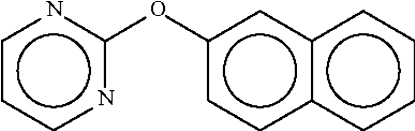


## Experimental

### 

#### Crystal data

C_14_H_10_N_2_O
*M* *_r_* = 222.24
Orthorhombic, 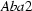

*a* = 13.0119 (3) Å
*b* = 22.4944 (5) Å
*c* = 7.5355 (2) Å
*V* = 2205.60 (9) Å^3^
*Z* = 8
Mo *K*α radiation
μ = 0.09 mm^−1^
*T* = 120 K
0.35 × 0.25 × 0.15 mm

#### Data collection

Bruker SMART APEX diffractometer
Absorption correction: none
7375 measured reflections
1366 independent reflections
1271 reflections with *I* > 2σ(*I*)
*R*_int_ = 0.026

#### Refinement

*R*[*F* ^2^ > 2σ(*F* ^2^)] = 0.029
*wR*(*F* ^2^) = 0.081
*S* = 1.03
1366 reflections
154 parameters
1 restraint
H-atom parameters constrained
Δρ_max_ = 0.19 e Å^−3^
Δρ_min_ = −0.18 e Å^−3^

### 

Data collection: *APEX2* (Bruker, 2008 ▶); cell refinement: *SAINT* (Bruker, 2008 ▶); data reduction: *SAINT*; program(s) used to solve structure: *SHELXS97* (Sheldrick, 2008 ▶); program(s) used to refine structure: *SHELXL97* (Sheldrick, 2008 ▶); molecular graphics: *X-SEED* (Barbour, 2001 ▶); software used to prepare material for publication: *publCIF* (Westrip, 2009 ▶).

## Supplementary Material

Crystal structure: contains datablocks global, I. DOI: 10.1107/S1600536809026592/bt2997sup1.cif

Structure factors: contains datablocks I. DOI: 10.1107/S1600536809026592/bt2997Isup2.hkl

Additional supplementary materials:  crystallographic information; 3D view; checkCIF report

## Figures and Tables

**Fig. 1.**
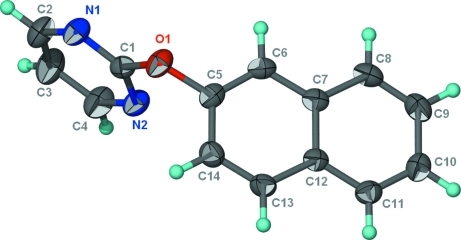
Anisotropic displacement ellipsoid plot (Barbour, 2001) of C14H10N2O at the 70% probability level; hydrogen atoms are drawn as spheres of arbitrary radius.

## supplementary crystallographic information

### Experimental

2-Naphthol (2.88 g, 20 mmol) and sodium hydroxide (0.80 g, 20 mmol) were dissolved in water (50 ml) and to the solution was added 2-chloropyridimidine (2.60 g, 20 mmol) dissolved in THF (50 ml). The mixture was heated for 4 h. Water was added and the organic phase was extracted with chloroform. The chloroform solution was dried over sodium sulfate; slow evaporation led to the formation of colorless crystals.

### Refinement

H-atoms were placed in calculated positions (C—H 0.95 Å) and were included in the refinement in the riding model approximation, with *U*(H) set to 1.2*U*(C).

In the absence of anomalous scatterers, 1111 Friedel pairs were merged and the absolute structure was arbitrarily set.

### Crystal data

**Table:**

C_14_H_10_N_2_O	*F*(000) = 928
*M**_r_* = 222.24	*D*_x_ = 1.339 Mg m^−^^3^
Orthorhombic, *A**b**a*2	Mo *K*α radiation, λ = 0.71073 Å
Hall symbol: A 2 -2ac	Cell parameters from 3105 reflections
*a* = 13.0119 (3) Å	θ = 2.4–28.0°
*b* = 22.4944 (5) Å	µ = 0.09 mm^−^^1^
*c* = 7.5355 (2) Å	*T* = 120 K
*V* = 2205.60 (9) Å^3^	Block, colorless
*Z* = 8	0.35 × 0.25 × 0.15 mm

### Data collection

**Table:**

Bruker SMART APEX diffractometer	1271 reflections with *I* > 2σ(*I*)
Radiation source: fine-focus sealed tube	*R*_int_ = 0.026
graphite	θ_max_ = 27.5°, θ_min_ = 1.8°
ω scans	*h* = −16→16
7375 measured reflections	*k* = −29→29
1366 independent reflections	*l* = −9→9

### Refinement

**Table:**

Refinement on *F*^2^	Primary atom site location: structure-invariant direct methods
Least-squares matrix: full	Secondary atom site location: difference Fourier map
*R*[*F*^2^ > 2σ(*F*^2^)] = 0.029	Hydrogen site location: inferred from neighbouring sites
*wR*(*F*^2^) = 0.081	H-atom parameters constrained
*S* = 1.03	*w* = 1/[σ^2^(*F*_o_^2^) + (0.0476*P*)^2^ + 0.7203*P*] where *P* = (*F*_o_^2^ + 2*F*_c_^2^)/3
1366 reflections	(Δ/σ)_max_ = 0.001
154 parameters	Δρ_max_ = 0.19 e Å^−^^3^
1 restraint	Δρ_min_ = −0.18 e Å^−^^3^

### Fractional atomic coordinates and isotropic or equivalent isotropic displacement parameters (Å^2^)

**Table:**

	*x*	*y*	*z*	*U*_iso_*/*U*_eq_	
O1	0.05750 (10)	0.07574 (6)	0.49878 (19)	0.0292 (3)	
N1	0.15301 (12)	0.04684 (7)	0.7306 (2)	0.0289 (4)	
N2	−0.00893 (12)	0.09426 (7)	0.7772 (2)	0.0282 (3)	
C1	0.06580 (13)	0.07234 (7)	0.6787 (2)	0.0225 (4)	
C2	0.16628 (15)	0.04528 (9)	0.9060 (3)	0.0343 (5)	
H2	0.2275	0.0280	0.9513	0.041*	
C3	0.09591 (17)	0.06737 (10)	1.0241 (3)	0.0394 (5)	
H3	0.1074	0.0663	1.1486	0.047*	
C4	0.00719 (16)	0.09122 (10)	0.9522 (3)	0.0362 (5)	
H4	−0.0442	0.1060	1.0302	0.043*	
C5	−0.02862 (13)	0.10606 (8)	0.4298 (2)	0.0247 (4)	
C6	−0.01790 (13)	0.16416 (8)	0.3824 (3)	0.0246 (4)	
H6	0.0460	0.1839	0.3985	0.030*	
C7	−0.10287 (13)	0.19501 (7)	0.3090 (2)	0.0233 (4)	
C8	−0.09853 (15)	0.25595 (8)	0.2640 (3)	0.0306 (4)	
H8	−0.0356	0.2770	0.2763	0.037*	
C9	−0.18366 (15)	0.28497 (8)	0.2030 (3)	0.0326 (4)	
H9	−0.1795	0.3260	0.1747	0.039*	
C10	−0.27770 (14)	0.25454 (8)	0.1818 (3)	0.0292 (4)	
H10	−0.3367	0.2753	0.1407	0.035*	
C11	−0.28390 (13)	0.19533 (8)	0.2202 (3)	0.0266 (4)	
H11	−0.3471	0.1749	0.2032	0.032*	
C12	−0.19730 (13)	0.16397 (7)	0.2850 (2)	0.0226 (3)	
C13	−0.20239 (14)	0.10272 (8)	0.3295 (3)	0.0271 (4)	
H13	−0.2641	0.0813	0.3086	0.033*	
C14	−0.11979 (14)	0.07416 (7)	0.4021 (3)	0.0277 (4)	
H14	−0.1241	0.0333	0.4333	0.033*	

### Atomic displacement parameters (Å^2^)

**Table:**

	*U*^11^	*U*^22^	*U*^33^	*U*^12^	*U*^13^	*U*^23^
O1	0.0250 (6)	0.0400 (7)	0.0225 (6)	0.0093 (5)	0.0003 (5)	−0.0022 (6)
N1	0.0250 (7)	0.0328 (8)	0.0290 (9)	0.0070 (6)	−0.0002 (6)	−0.0046 (7)
N2	0.0237 (7)	0.0350 (8)	0.0260 (8)	0.0053 (6)	0.0005 (7)	−0.0019 (7)
C1	0.0219 (8)	0.0222 (7)	0.0235 (9)	−0.0017 (6)	−0.0003 (7)	−0.0034 (7)
C2	0.0309 (10)	0.0381 (10)	0.0339 (12)	0.0091 (8)	−0.0079 (9)	−0.0034 (8)
C3	0.0435 (12)	0.0513 (13)	0.0234 (11)	0.0129 (10)	−0.0051 (9)	−0.0014 (9)
C4	0.0331 (10)	0.0500 (12)	0.0255 (11)	0.0115 (9)	0.0047 (9)	−0.0026 (9)
C5	0.0227 (8)	0.0335 (9)	0.0180 (8)	0.0034 (7)	0.0001 (7)	−0.0016 (7)
C6	0.0198 (8)	0.0323 (8)	0.0218 (9)	−0.0040 (6)	0.0013 (7)	−0.0037 (7)
C7	0.0245 (8)	0.0275 (8)	0.0177 (8)	−0.0040 (7)	0.0020 (7)	−0.0029 (7)
C8	0.0296 (9)	0.0299 (9)	0.0322 (10)	−0.0082 (7)	0.0042 (8)	−0.0005 (8)
C9	0.0380 (10)	0.0240 (8)	0.0358 (11)	−0.0032 (7)	0.0041 (10)	0.0029 (8)
C10	0.0300 (9)	0.0314 (8)	0.0263 (10)	0.0050 (7)	−0.0013 (8)	0.0007 (8)
C11	0.0236 (8)	0.0318 (8)	0.0243 (9)	−0.0029 (6)	−0.0013 (7)	−0.0005 (7)
C12	0.0231 (8)	0.0261 (8)	0.0186 (8)	−0.0032 (6)	0.0007 (7)	−0.0011 (7)
C13	0.0254 (8)	0.0275 (8)	0.0285 (10)	−0.0066 (7)	−0.0034 (8)	0.0012 (7)
C14	0.0293 (9)	0.0236 (8)	0.0303 (11)	−0.0007 (7)	0.0006 (9)	0.0009 (7)

### Geometric parameters (Å, °)

**Table:**

O1—C1	1.362 (2)	C7—C8	1.413 (2)
O1—C5	1.411 (2)	C7—C12	1.425 (2)
N1—C1	1.330 (2)	C8—C9	1.365 (3)
N1—C2	1.334 (3)	C8—H8	0.9500
N2—C1	1.319 (2)	C9—C10	1.411 (3)
N2—C4	1.337 (3)	C9—H9	0.9500
C2—C3	1.370 (3)	C10—C11	1.365 (3)
C2—H2	0.9500	C10—H10	0.9500
C3—C4	1.383 (3)	C11—C12	1.416 (2)
C3—H3	0.9500	C11—H11	0.9500
C4—H4	0.9500	C12—C13	1.420 (2)
C5—C6	1.362 (2)	C13—C14	1.366 (3)
C5—C14	1.402 (2)	C13—H13	0.9500
C6—C7	1.418 (2)	C14—H14	0.9500
C6—H6	0.9500		
			
C1—O1—C5	117.18 (14)	C6—C7—C12	118.81 (14)
C1—N1—C2	114.41 (16)	C9—C8—C7	120.79 (16)
C1—N2—C4	114.86 (16)	C9—C8—H8	119.6
N2—C1—N1	128.65 (17)	C7—C8—H8	119.6
N2—C1—O1	118.73 (15)	C8—C9—C10	120.66 (16)
N1—C1—O1	112.61 (15)	C8—C9—H9	119.7
N1—C2—C3	123.22 (19)	C10—C9—H9	119.7
N1—C2—H2	118.4	C11—C10—C9	120.02 (17)
C3—C2—H2	118.4	C11—C10—H10	120.0
C2—C3—C4	116.4 (2)	C9—C10—H10	120.0
C2—C3—H3	121.8	C10—C11—C12	120.80 (16)
C4—C3—H3	121.8	C10—C11—H11	119.6
N2—C4—C3	122.46 (19)	C12—C11—H11	119.6
N2—C4—H4	118.8	C11—C12—C13	121.86 (16)
C3—C4—H4	118.8	C11—C12—C7	119.05 (14)
C6—C5—C14	122.60 (16)	C13—C12—C7	119.08 (16)
C6—C5—O1	118.62 (16)	C14—C13—C12	120.94 (16)
C14—C5—O1	118.65 (15)	C14—C13—H13	119.5
C5—C6—C7	119.48 (15)	C12—C13—H13	119.5
C5—C6—H6	120.3	C13—C14—C5	118.99 (15)
C7—C6—H6	120.3	C13—C14—H14	120.5
C8—C7—C6	122.49 (15)	C5—C14—H14	120.5
C8—C7—C12	118.66 (16)		
			
C4—N2—C1—N1	1.5 (3)	C6—C7—C8—C9	−176.20 (19)
C4—N2—C1—O1	−177.46 (18)	C12—C7—C8—C9	1.6 (3)
C2—N1—C1—N2	−2.1 (3)	C7—C8—C9—C10	−0.7 (3)
C2—N1—C1—O1	176.91 (17)	C8—C9—C10—C11	−0.8 (3)
C5—O1—C1—N2	3.8 (2)	C9—C10—C11—C12	1.3 (3)
C5—O1—C1—N1	−175.36 (14)	C10—C11—C12—C13	178.62 (19)
C1—N1—C2—C3	0.7 (3)	C10—C11—C12—C7	−0.3 (3)
N1—C2—C3—C4	1.0 (3)	C8—C7—C12—C11	−1.1 (3)
C1—N2—C4—C3	0.5 (3)	C6—C7—C12—C11	176.80 (17)
C2—C3—C4—N2	−1.7 (4)	C8—C7—C12—C13	179.90 (17)
C1—O1—C5—C6	96.5 (2)	C6—C7—C12—C13	−2.2 (3)
C1—O1—C5—C14	−87.6 (2)	C11—C12—C13—C14	−175.94 (19)
C14—C5—C6—C7	2.8 (3)	C7—C12—C13—C14	3.0 (3)
O1—C5—C6—C7	178.52 (16)	C12—C13—C14—C5	−1.0 (3)
C5—C6—C7—C8	177.22 (18)	C6—C5—C14—C13	−2.0 (3)
C5—C6—C7—C12	−0.6 (3)	O1—C5—C14—C13	−177.73 (17)

## References

[bb1] Barbour, L. J. (2001). *J. Supramol. Chem.* **1**, 189–191.

[bb2] Bruker (2008). *APEX2* and *SAINT* Bruker AXS Inc., Madison, Wisconsin, USA.

[bb3] Shah Bakhtiar, N., Abdullah, Z. & Ng, S. W. (2009). *Acta Cryst.* E**65**, o114. 10.1107/S1600536808041196 PMC2968036 21581576

[bb4] Sheldrick, G. M. (2008). *Acta Cryst.* A**64**, 112–122. 10.1107/S0108767307043930 18156677

[bb5] Westrip, S. P. (2009). *publCIF* In preparation.

